# Cultivar Resistance against *Colletotrichum asianum* in the World Collection of Mango Germplasm in Southeastern Brazil

**DOI:** 10.3390/plants9020182

**Published:** 2020-02-02

**Authors:** Alessandro Vitale, Acelino Couto Alfenas, Dalmo Lopes de Siqueira, Donato Magistà, Giancarlo Perrone, Giancarlo Polizzi

**Affiliations:** 1 Dipartimento di Agricoltura, Alimentazione e Ambiente, Università di Catania, 95123 Catania, Italy; gpolizzi@unict.it; 2 Laboratory of Forest Pathology/Bioagro, Departamento de Fitopatologia, Universidade Federal de Viçosa, Viçosa 36570-900, Brazil; aalfenas@ufv.br; 3 Departamento de Fitotecnia, Universidade Federal de Viçosa, Viçosa 36570-900, Brazil; siqueira@ufv.br; 4 Istituto di Scienze delle Produzioni Alimentari, Consiglio Nazionale delle Ricerche (ISPA-CNR), 70126 Bari, Italy; donato.magista@ispa.cnr.it (D.M.); giancarlo.perrone@cnr.it (G.P.)

**Keywords:** anthracnose, *Mangifera indica*, morphological characterization, phylogenetic analyses, disease tolerance

## Abstract

During the spring of 2014, a wide survey was conducted in one of the most important mango (*Mangifera indica*) cultivating areas located in Minas Gerais State (Brazil) to ascertain the causal agent of severe anthracnose infections and to evaluate disease susceptibility within a world collection of mango germplasm. Overall, 86 cultivars were monitored and 152 fungal isolates recovered from infected samples were identified by morphological characterization, DNA sequencing and phylogenetic analyses. All isolates were identified as *Colletotrichum asianum*. Under natural disease pressure, it has been possible to ascertain a variable tolerance degree within the germplasm collection. By applying a categorized classification, cultivars were classified as follows: 10 highly sensitive (11.6%), 13 sensitive (15.1%), 18 moderately sensitive (20.9%), 23 moderately tolerant (26.7%), 11 tolerant (12.8%), and 11 highly tolerant (10.4%). The most susceptible cultivars to anthracnose were Ubà, Quinzenga, Amarelinha da Sementeira followed by Aroeira and Correjo, whereas Mallika followed by Ourinho and Lita resulted in the least susceptible cultivars. To the authors’ knowledge, this is the first large-scale evaluation of mango susceptibility to *C. asianum* infections within a wide number of cultivars. Anthracnose is a serious threat to mango production and assessment of cultivar response to disease could be useful in breeding programs.

## 1. Introduction

Mango (*Mangifera indica* L.) is the predominant tropical fruit in the world being cultivated in more than one hundred countries and accounting for more than half of global major tropical fruit production [1]. Currently, about 80% of global production is concentrated in nine nations. Brazil is the 7th largest producer in the world [2]. Mango is sometimes referred to as the king of the fruits, due to its eye-catching color, pleasant taste, the existence of higher concentrations of carotenoids, ascorbic acid and phytochemicals [3]. About 80% of the commodity is consumed as such; while 20% is processed into value-added products, such as mango puree, nectar, pickles, chutneys and canned products [4]. Unfortunately, infections caused by several pathogen species belonging to the *Colletotrichum* genus represent the most serious threat for mango cultivation worldwide. Disease caused by these fungal pathogens can negatively impact both yield and fruit quality [5,6].

Up to 2009 *Colletotrichum gloeosporioides s. lat.* had been considered the main causal agent of mango anthracnose followed to lesser extent by *C. acutatum* [5,6,7,8]. Since the multi-gene phylogenetic analysis and poly-phasic approach were adopted, the ability to distinct phylogenetic (cryptic) species within *Colletotrichum* genus [9,10,11,12,13,14], including *C. gloeosporioides* [14,15,16,17,18,19,20] was strongly improved. Taxonomic revisions have resulted in the identification of several new species that include *C asianum, C. dianesei, C. fructicola, C. siamense, C. tropicale* and *C. karstii* that are pathogenic on mango [14,21,22,23,24]. Other species belonging to *C. acutatum s. lat.* are also know to cause anthracnose on mango plantations. 

Infections result in irregularly shaped, black necrotic spots on upper and lower leaf surfaces. Lesions frequently coalesce to form large necrotic areas, and severely infected leaves often curl. Leaf spots develop mainly on young tissue whereas in senescent leaf tissues the infections are not visible and the fungus remains dormant (latent infections). Symptoms can sometimes be observed as twig dieback, stalk lesions and flower blight. Although many portions of the plant may be infected, the major losses occur during ripening and in postharvest when brown or black lesions developed on fruit surfaces [5,25,26].

Among all these species *C. asianum* is one of the most common and representative species associated with mango anthracnose, being reported from Australia, Brazil, China, Colombia, Ghana, India, Japan, Malaysia, Mexico, Panama, Philippines, South Africa, Sri Lanka, Thailand and Florida [14,18,20,21,22,27,28,29,30,31,32]. 

The objectives of this study were: (i) to identify the *Colletotrichum* spp. associated with mango anthracnose using morphological, molecular characterization and multi-gene phylogenetic analysis; (ii) to compare the susceptibility of cultivars in southeastern Brazil to anthracnose infections; and (iii) to identify the cultivars representative in each of the susceptibility groups. Phenotypic evaluation of susceptibility to the pathogen under field conditions could be very useful for the selection of mango cultivars by farmers, technicians, and breeders.

## 2. Results

### 2.1. Morphological Characterization 

**Figure 1:**
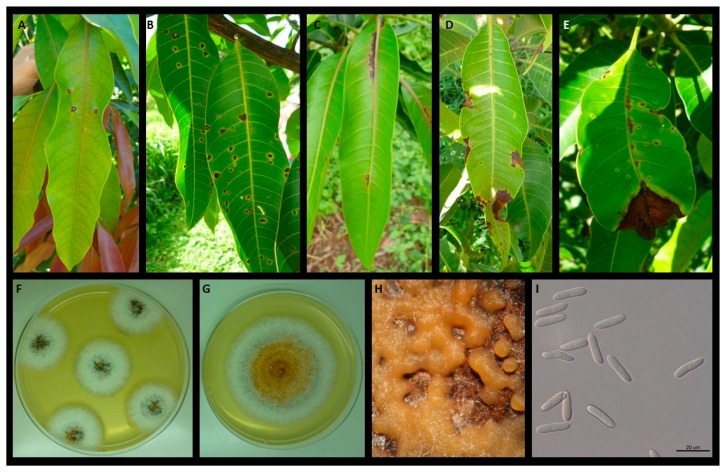
Variability in disposal, patterns and size on leaf blade of anthracnose infections caused by *Colletotrichum asianum* during susceptibility mango evaluation at the Sementeira Farm, Universidade Federal de Viçosa (**A**–**E**). Culture characteristics and microscopic features of the *C. asianum*: colony morphology from isolation attempts (**F**), 10-day-old monoconidial isolate (**G**), conidiomata on host tissues (**H**) and conidia (**I**).

A total of 152 monoconidial *Colletotrichum* isolates were collected from all evaluated mango cultivars showing leaf symptoms different in disposal, size and shape on the leaf blade (Figure 1A–E). Colonies grown on PDA were at first white-orange, then turned to greenish-grey to dark green at the center with the age. On the reverse side, colonies appear dark green at the center. The mean daily growth rate at 25 °C ranged from 4.0 to 5.1 mm. Colonies produced aerial mycelium in small tufts, white, sparse, with orange to dark orange conidial masses. The length and width of conidia produced by *Colletotrichum* isolates ranged from 10.5 to 19.7 μm and 3.2 to 4.5 μm, respectively, and they were common in mycelium and conidial masses. They were one-celled, smooth-walled, guttulate, hyaline, cylindrical with obtuse ends (oblong) with slight narrowing at the center (Figure 1F–I). Sclerotia, acervuli and setae were absent in culture. All these characteristics are in accordance to those reported for *C. asianum* [33].

### 2.2. Phylogenetic Analysis

**Figure 2:**
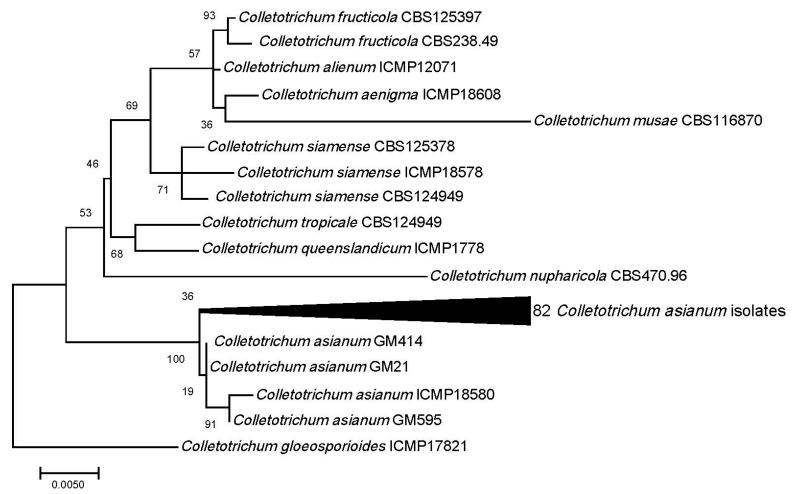
Three loci (ITS, TUB, HIS) phylogeny of the 82 *C. asianum* isolated from *Mangifera indica* in the Brazilian Mango Germplasm Collection at the Sementeira Farm, Universidade Federal de Viçosa in southeastern Brazil.

From the phylogenetic analysis of the tree loci (ITS, TUB, HIS) considered in this study, all of the 82 *Colletotrichum* isolates, randomly selected from 152 isolates previously morphologically characterized, belong to *C. asianum*. Two other species (*C. aeschynomenes* and *C. salsolae*) of the *C. gloeosporioides* clade *musae*, were not included in the tree loci phylogeny, since no HIS sequence was available (Figure 2). However, these two species, were not close to *C. asianum*, thus, can be excluded from the three loci analysis. Moreover, the two loci analysis (data not shown) confirm that they were distinct from *C. asianum*. There were a total of 1255 positions in the final dataset of the combined three loci. The tree with the highest log likelihood (-2531.80) is shown in Figure 2, the tree is drawn to scale, with branch lengths measured in the number of substitutions per site, and the percentage of trees in which the associated taxa clustered together is shown next to the branches (bootstraps).

### 2.3. Susceptibility of Mango Cultivars

The amount of anthracnose leaf infection varied among accessions during the crop season. Due to the high number of detected cultivars, the data showed a continuous gradation in the pathogen susceptibility ranging from high sensitivity to strong tolerance. In other words, the anthracnose susceptibility ranged from mango cultivars with very few symptomatic leaves to cultivars with all infected leaves. Since *Colletotrichum* is also able to induce leaf anthracnose infections very variable in the number of spots, size and placement on leaf blade (Figure 1A–E) an empirical method has been set up for grouping mango accessions taking simultaneously into account DI and SS value (Table 1).

**Figure 3:**
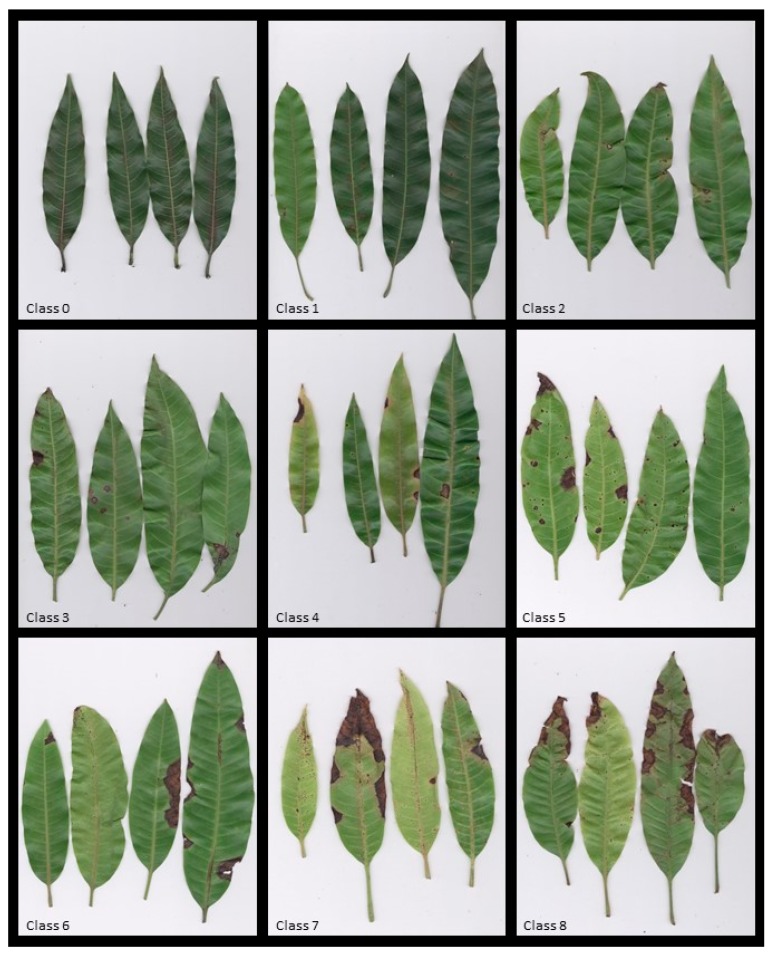
Nine (0-to-8) of total (13) disease classes detected within empirical scale adopted to evaluate severity of leaf infections caused by *C. asianum* during susceptibility mango evaluation at the Sementeira Farm, Universidade Federal de Viçosa (A–E).

Disease incidence was calculated on the basis of the percentage of symptomatic leaves on each plant, whereas SS always referred to an empirical 0-to-12 rating scale set-up properly for the evaluation of leaf anthracnose amount as well explained in the materials and methods section (Figure 3). 

Subsequently, the average DI and SS data relative to each phenotype group are reported in Table 2. 

No resistant cultivars were present in this germplasm collection. Among them, 11 were classified as strongly tolerant since DI was always less than 9% and SS index ranging from 1 to 2. The same number (11) were categorized as tolerant with DI ranging from 9.5% to 18.75% and mean SS higher than 2 (Table 2). On the other hand, 10 and 13 accessions, intercepting mean DI values of about 97% and 65%, and SS of about 5.8 and 4.6, were classified as highly sensitive and sensitive, respectively. The remaining 41 cultivars were classified as moderately tolerant (23) and moderately sensitive (18) with intermediate DI and SS values as it is reported in Table 2. 

**Figure 4:**
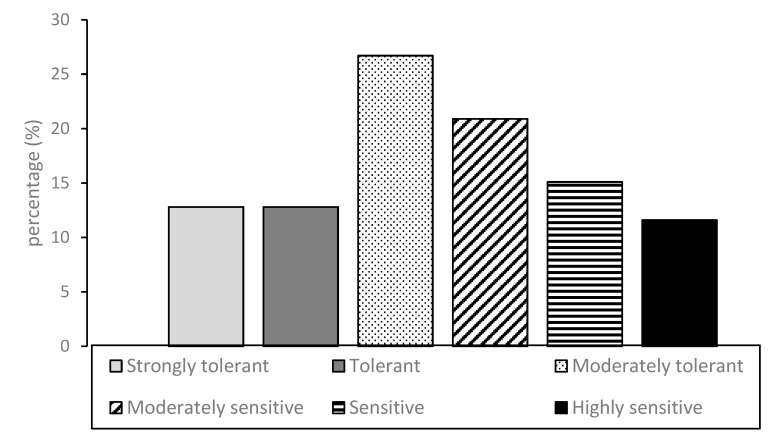
Percentage distribution of mango cultivars having a different sensitivity/tolerance response to anthracnose leaf infections caused by *C. asianum* in the Brazilian Mango Germplasm Collection at the Sementeira Farm, Universidade Federal de Viçosa.

However, the frequency distribution of mango cultivars within the established categories is not perfectly balanced (roughly normal distribution). As expected, it shows a prevalence of intermediate susceptibility categories (moderately tolerant and moderately sensitive) with a cumulative value of 47.7% on the total of examined germplasm whereas the distribution tails intercept 26.7% of susceptible (sensitive and highly sensitive) and 25.6% of resistant (tolerant and highly tolerant) accessions, respectively (Figure 4).

**Figure 5:**
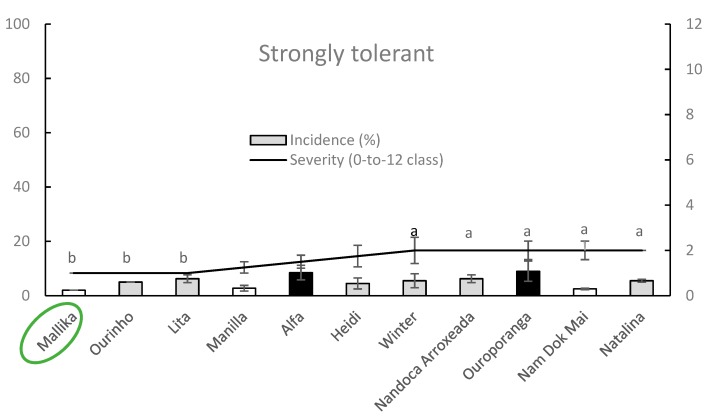
Column-line graphs on two axes comparing anthracnose DI and SS caused by *C. asianum* within strongly tolerant mango cultivars. Average DI and SS data from three monitoring times (± SE: standard error) are the means of four replicates (plants) obtained from disease incidence on each canopy and 16 leaves per plant, respectively. Arcsine transformation was used on percentage data prior to analysis, whereas untransformed data are presented. Black and white columns are significantly different among them according to Fisher’s least significant difference test at *α* = 0.05 while grey color denotes intermediate behavior (not significant). Differences among severity data on-line among cultivars (points followed by different letters) were analyzed with Kruskal–Wallis one-way analysis of variance by ranks followed by all pairwise multiple comparisons with Mann–Whitney test. The missing letters denote non-significant differences from all remaining severity values.

Within strongly tolerant germplasm group, Mallika, Manilla and Nam Dok Mai showed significantly lower DI values if compared with Alfa and Ouroporanga cultivars whereas the SS values were significantly lower on Mallika, Ourinho and Lita cultivars if compared with those observed on Winter Nandoca Arroxeada, Ouroporanga, Nam Dok Mai and Natalina. Comprehensively, Mallika exhibited the lowest susceptibility to leaf anthracnose infections incited by *C. asianum* while Ouroporanga displayed the highest disease amount values (Figure 5). 

**Figure 6:**
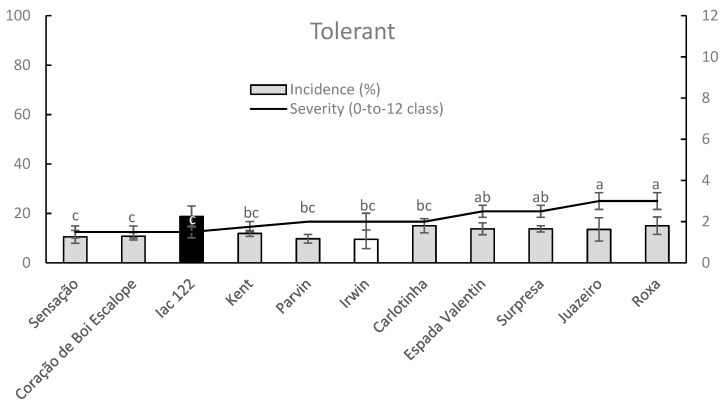
Column-line graphs on two axes comparing anthracnose DI and SS caused by *C. asianum* within tolerant mango cultivars. Data (± SE values) were collected and analyzed as above mentioned for the previous figure. Grey color of columns clearly shows the lack of significant differences according to both parametric and nonparametric approaches.

Irwin cultivar showed a DI value significantly lower than one observed on Iac122 cultivar within tolerant mango germplasm group whereas the remaining cultivars showed an intermediate anthracnose incidence. Regarding the mean SS data, Sensação, Coração de Boi Escalope, and Iac122 significantly differed (lower data) from Juazeiro and Roxa cultivars whereas Kent (among the most well-known cultivars), Irwin (with a low disease incidence) and remaining tolerant cultivars had intermediate leaf anthracnose severities (data not significant) (Figure 6). 

**Figure 7:**
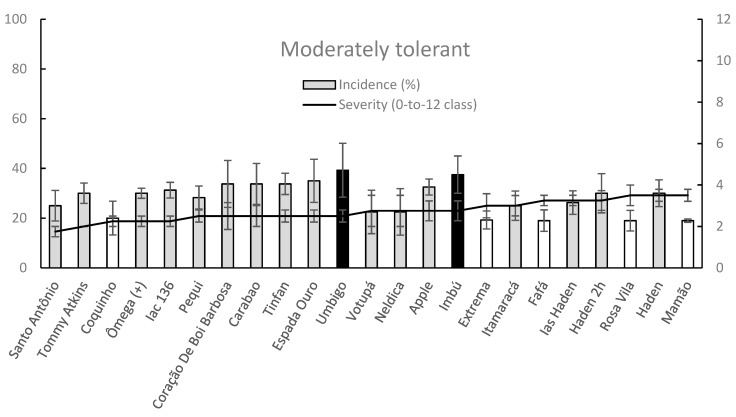
Column-line graphs on two axes comparing anthracnose incidence and severity caused by *C. asianum* within moderately tolerant mango cultivars. Data (± SE values) were collected and analyzed as above done for previous figures. Grey colors of columns, as well as the absence of letters above the points of line, clearly show the lack of significant differences according to both parametric and nonparametric approaches.

**Figure 8:**
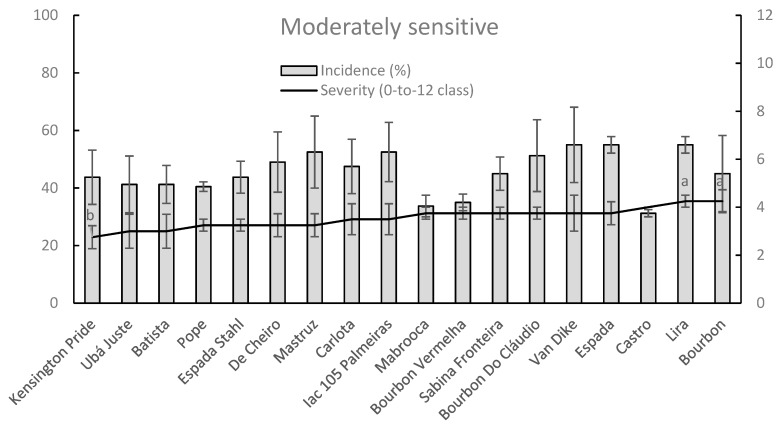
Column-line graphs on two axes comparing anthracnose incidence and severity caused by *C. asianum* within moderately sensitive mango cultivars. Data (± SE values) were collected and analyzed as above done for previous figures. Grey colors of columns, as well as the absence of letters above points of the line, clearly show the lack of significant differences according to both parametric and nonparametric approaches.

Since a great variability was investigated on mango orchard for DI and SS parameters within moderately tolerant and moderately sensitive groups respectively it was very difficult to discriminate significant differences among examined cultivars within these groups, respectively (Figure 7 and Figure 8). 

In the moderately tolerant group, only Couquinho, Extrema, Fafá, Rosa Vila and Mamão showed DI values significantly lower than those of Umbigo and Imbú whereas the remaining ones, including the well-known mango cultivars Tommy Atkins and Haden (Figure 7).

Otherwise, within the moderately sensitive group the only significant differences amongst cultivars were detected for SS parameter between well-known Kensington Pride (lower values) and Lira and Bourbon (higher values) (Figure 8).

**Figure 9:**
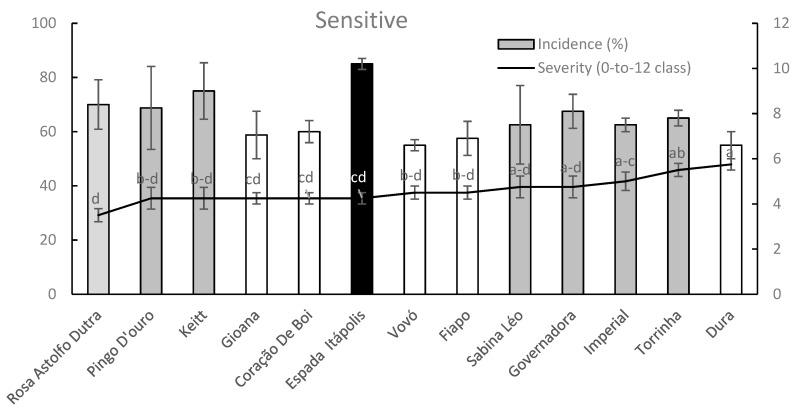
Column-line graphs on two axes comparing anthracnose incidence and severity caused by *C. asianum* within sensitive mango cultivars. Data (± SE values) were collected and analyzed as above done for previous figures. Grey colors of columns, as well as the absence of letters above points of the line, clearly shows the lack of significant differences according to both parametric and nonparametric approaches.

Within sensitive mango germplasm group (Figure 9), five cultivars (Gioana, Coração de Boi, Vovό, Fiapo and Dura) exhibited a significant lower anthracnose diffusion (DI) data when compared with Espada Itápolis cultivar while the remaining cultivars did not significantly differ among all tested cultivars. 

On the other hand, Rosa Astolfo Dutra exhibited a mean SS value significantly lower than those of Imperial, Torrinha and Dura whereas intermediate anthracnose sensitivity values were recorded for remaining cultivars including the well-known cultivar Keitt (Figure 9). 

**Figure 10:**
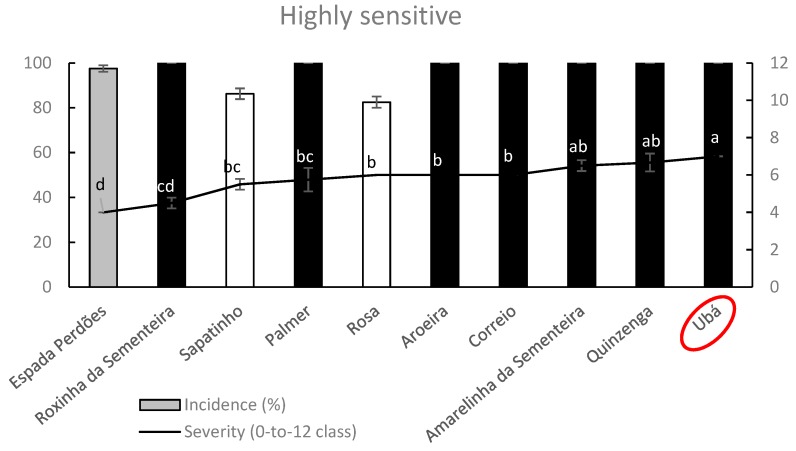
Column-line graphs on two axes comparing anthracnose incidence and severity caused by *C. asianum* within highly sensitive mango cultivars. Data (± SE values) were collected and analyzed as above done for previous figures. Grey colors of columns, as well as the absence of letters above points of the line, clearly shows the lack of significant differences according to both parametric and nonparametric approaches.

In the highly sensitive germplasm group (Figure 10), mango cultivars Sapatinho and Rosa showed anthracnose incidence values significantly lower (α = 0.05) than the remaining cultivars except for the Espada Perdões (grey color). On the other hand, this latter cultivar exhibited an SS value significantly lower than those of all cultivars of the group except for Roxinha da Sementeira. 

Overall, cultivar Ubá followed by Quinzenga and Amarelinha da Sementeira resulted in higher sensitivity to anthracnose infections caused by *C. asianum.* Other well-known mango cultivar Palmer fell in this highly sensitive germplasm group (Figure 10).

## 3. Discussion

In this study, we present the first data on the evaluation of susceptibility to anthracnose leaf infections in an important mango germplasm collection of southeastern Brazil. The paper also represents the first report describing *Colletotrichum* species responsible for the widespread occurrence of anthracnose infections on mango orchards in the Minas Gerais region. *C. asianum*, *C. dianesei*, *C. fructicola*, *C. karstii*, *C. tropicale*, *C. cliviae* and *C. endomangiferae* have been previously reported as responsible for mango anthracnose in Brazil [21,24,34], whereas, in the present study both morphological and phylogenetic analyses revealed that solely *C. asianum* was found associated with disease infections in field. In particular, the three genes phylogeny clearly grouped the 82 representative strains within the *C. asianum* branch with high bootstrap support (100%). This finding is not surprising since this latter species is worldwide reported as the most prevalent and virulent from the main mango production areas of Brazil and around the world [24,28,30,31,32,34,35]. However, the presence of a single species as a causal agent of anthracnose has facilitated the disease susceptibility assessment when compared to other phytosanitary conditions where more *Colletotrichum* species are involved in disease infections. Although the disease evaluation was done on mango plant canopies (leaves), these data are valuable since the fungus is polycyclic and the role exerted by leaf infections in enhancing disease amount and duration of epidemics is crucial [36]. This is more evident in mango since this crop has at least three vegetative periods, depending on adopted cultivar [37,38]. However, it well-known as early leaf infection assessment can represent an efficient predictive method to reduce fruit losses in the orchard [39,40,41] as it was demonstrated for mango germplasm screening to anthracnose where leaf and fruit infections coexisted in orchard [32,42,43,44]. Although cultivar response to anthracnose attacks was very variable there is a clear predominance of cultivars with intermediate tolerance/susceptibility degrees, i.e., with moderate tolerance and moderate susceptibility toward anthracnose. As a consequence, a clear separation of susceptibility behavior within these groups was not always possible amongst these mango accessions as it happens for Tommy Atkins and Haden, and Kensington Pride that were assigned to these groups. Currently, resistance has not been used as consistent means for control of mango anthracnose. This is partially due to the variable disease response of cultivars to the anthracnose from one location to another. In this regard, the literature on host resistance of mango germplasm reported worldwide are quite controversial [42,45,46,47,48,49,50,51]. Besides to above reason, this discrepancy is also due to the fact that local susceptibility evaluations were always referred to *C. gloeosporioides s. lat*. without considering accurate *Colletotrichum* species involved in anthracnose infections. It is also interesting note that none of the accessions examined here were resistant and none of the commercial cultivars cannot generally provide under environment humid conditions adequate qualitative and quantitative yields without scheduled fungicide spray applications [5]. To sum up, Mallika revealed in our phytosanitary conditions to be the most tolerant accession to *C. asianum* attacks, while Kent and Irwin were categorized as tolerant accessions. On the other hand, the well-known cultivar Ubá for this Brazilian region revealed to be the most sensitive to fungal infections of *C. asianum*. The well-known cultivar Keitt revealed to be sensitive to anthracnose attacks.

Although these findings should be confirmed under different conditions, the paper gained for the first time a preliminary insight about the susceptibility of several mango species to *C. asianum*. The establishment of susceptibility to *C. asianum* against which coming changes can be measured is a crucial and starting point for evaluating the tolerance of local, commercial and well-known mango germplasm under specific agronomic and phytosanitary conditions. For future studies, the methodology described here should also be used to evaluate the cultivar response to other aerial fungal pathogens affecting canopy of mango. Although host resistance alone is not resolutive, it can be considered a sustainable mean since pathologists and breeders can use it both to implement IPM strategies for mango anthracnose caused by *C. asianum* and to reduce number fungicide treatments.

## 4. Materials and Methods

### 4.1. Plant Material and Orchard

A wide disease survey was performed in 2014 using the most important mango germplasm collection located in Southeastern Brazil (Minas Gerais, MG region). The entire collection constitutes about two hundred accessions of mango cultivars from different countries and areas of origin. The accessions are conserved as a live collection at the Sementeira Farm located in a wide orchard subdivided into several areas. The entire collection (Banco De Germoplasma De Mangueiras, Fazenda Experimental da Sementeira—BGM-FES), belonging to the Universidade Federal de Viçosa in the municipality of Visconde do Rio Branco, MG (21º00′37”S, 42º50′26”W; 352 m altitude), had in the recent past growing seasons a history of severe anthracnose infections. For most cultivars tested in this paper, the degree susceptibility (with particular reference to strongly tolerant and highly sensitive cultivars) versus anthracnose was very similar to one presented here.

### 4.2. Isolations and Morphological Characterization

Isolations were made from symptoms of infected leaves of all cultivars. Small sections from the edge of lesions were disinfested in 1% NaOCl (1 min), rinsed in sterile distilled water (SDW) and placed onto potato dextrose agar (PDA, Oxoid, Basingstoke, UK) with streptomycin sulfate (100 µg/mL) and incubated at 25 ± 1 °C with a 12-h photoperiod. At least two single-spore isolates were obtained from each mango sample. Morphological characterization of isolates was performed by using single conidial cultures prepared on PDA. In detail, monoconidial cultures of *Colletotrichum* species were incubated at 25 °C in the dark on PDA. Conidia were examined after 10 days of incubation. Conidial shape and color were determined by mounting fungal structures in clear lactic acid. Measurements of 50 conidia for each representative isolate were determined at 400 and 1000× using an optical microscope with interference contrast illumination. The mean measurements of conidia were calculated. Colony characteristics were determined after seven-day period growth at 25 °C on PDA.

### 4.3. DNA Isolation, PCR and Phylogeny

The species-level identification was obtained by DNA sequencing and phylogenetic analyses of β-tubulin (TUB2), histone H3 (HIS3) and nuclear ribosomal internal transcribed spacer (ITS) region gene sequences of the 82 strains (almost one representative isolate from each examined cultivar) tested in this study. Isolates were grown in potato dextrose broth (PDB, Sigma-Aldrich, Saint Louis, MO, USA) incubated at 25 °C in the dark, under shaking, for 4-6 days. Mycelial mats were collected, dried with sterile filter paper, frozen in liquid nitrogen and ground to a fine powder. Genomic DNA extraction was performed using Wizard Magnetic DNA Purification System for Food kit (Promega, Madison, WI, USA) according to the manufacturer’s protocol. The quality of genomic DNA was determined by agarose gel electrophoresis and quantified through a Nanodrop ND-1000 Spectrophotometer (Thermo Fisher Scientific, Wilmington, DE, USA). Part of the β-tubulin gene was amplified using primers T1 [52]) and Bt2b [53]. ITS1 and ITS4 primers [54] were used to amplify part of the Internal Transcribed Spacer (ITS) region of the rRNA (the 3′ end of the 18S rRNA gene, the internal spacers, the 5.8S rRNA gene and a part of the 5′ end of the 28S rRNA) gene. For the histone H3 region, CYLH3F and CYLH3R primers [55] were used. PCR amplifications were carried out with HotMaster Taq DNA Polymerase, nucleotides and buffer supplied by 5Prime (PRIME GmbH, Hamburg, Germany). The PCR reaction mixture contains 1x HotMaster Taq Buffer with Mg2+, 200 uM dNTP Mix, 1 U HotMaster Taq DNA Polymerase, 0,1uM of each primer and 50-100 ng of template DNA. Sequencing was performed using an AB 3730 DNA analyzer (Applied Biosystems, Foster City, CA, USA). BigDye Terminator cycle sequencing kit (version 3.1; Applied Biosystems) was used following the manufacturer’s manual on both strands by the same primers. The basic local alignment search tool (BLAST) in GenBank (www.ncbi.nlm.nih.gov/blast) was queried after aligning, editing and trimming the sequences by Geneious R10 (Biomatters Ltd.). Sequences of the three loci (TUB2, HIS3, ITS) of reference type strains belonging to the musae clade of the *C. gloeosporioides* species complex, were retrieved from GenBank. The multi-locus alignment was performed using MEGA-X [56] software with manual adjustment. The phylogenetic analyses were conducted on the combined multilocus alignment of two (ITS, TUB2) or three loci. Tamura [57] and Tamura-Nei [58] were the best evolution model suggested for the analysis of the two and three loci alignment, respectively. Phylogenetic analyses were inferred by using the maximum likelihood method with five gamma categories and 1000 bootstrap replications.

### 4.4. Monitoring and Field Sampling

During the crop season of 2014, 86 mango cultivars were evaluated in three different monitoring times (from April to May with an interval of 25 days) at the same locations for their susceptibility to leaf anthracnose infections under natural anthracnose disease pressure. Since low disease level, data on fruit infections collected during other months are not detected. Tested mango cultivars located in four distinct areas of germplasm collection were scored for their susceptibility/tolerance to leaf anthracnose infection evaluating four plants (four replicates) by assessment of disease incidence (DI) and symptoms severity (SS) parameters. The former (DI—qualitative parameter) was always referred to the assessment of average percentage of symptomatic leaves on entire canopy of each plant whereas SS (quantitative parameter) was accounted on 16 leaves (for each replicate of each cultivar, four leaves per each of four sub-replicates) adopting an empirical 0-to-12 rating scale set up for evaluation of leaf anthracnose amount. This empirical scale takes into account the mean percentage of infected surface where 0 = no symptoms; 1 = up to 0.5% of infected leaf surface; 2 = 0.6 to 1%; 3 = 1.1 to 2%; 4 = 2.1 to 3%; 5 = 3.1 to 4%; 6 = 4.1 to 6%; 7 = 6.1 to 10%; 8 = 10.1 to 25%; 9 = 25.1 to 40%; 10 = 40.1 to 60%; 11 = 60.1 to 75%; 12 = more than 75% of infected leaf surface. To measure the infected leaf area, mango leaves were well extended and the relative images were captured using a scanner (HP Scanjet G2710) (Figure 3). From the images, the infected leaf area was measured by ImageJ [59]. Definitively, the average leaf anthracnose severity was always calculated by the following formula:

$$SS=\frac{\sum_{i=0}^{12}\left(Ci\times n\right)}{N},$$

where SS is the average index of severity symptoms, C*i* each class detected, n the number of leaves in each class, *i* (0-to-12) the numerical values of classes, N the total number of leaves examined. The definitive DI and SS data (as average resulting from three evaluating periods since monitoring was carried out in triplicate) was always confirmed by an adequate number of isolation attempts performed in the laboratory (at least from 10 to 20 attempts for each leaf sample with little clear symptoms) from symptomatic mango samples (Figure 1F) and consequentially adjusted on the basis of obtained recovery data. Finally, the cultivars were classified into seven categories (resistant, strongly tolerant, tolerant, moderately tolerant, moderately susceptible, susceptible and highly susceptible) according to combined DI and SS definitive data.

### 4.5. Statistical Analysis

STATISTICA package software (version 10; Statsoft Inc., Tulsa, OK, USA) was used for statistical analyses according to parametric or nonparametric approaches for randomized complete block design (RCBD) with the different cultivars having four replicates. In the posthoc analysis the mean separation was conducted on DI data using post-hoc Fisher’s least significant difference test at α = 0.05. Prior to analysis, percentage values were transformed as arcsine square root (sin^−1^ square root x) to improve homogeneity of variances [60], whereas untransformed arithmetic means of DI are shown in the figures. Because an ordinal scale (0-to-12 empirical classes) was adopted for assessment of anthracnose severity (Figure 3), rank sums of SS data were analyzed according to a nonparametric approach, i.e., Kruskal-Wallis one-way analysis (χ^2^ value and associated p level < 0.05 indicate the significance) for experiment wise significance followed by all possible pairwise comparisons using the Mann-Whitney test (*z* > 2.58; *p* < 0.01) [61].

## Figures and Tables

**Table 1:** Phenotype groups of mango susceptibility according to anthracnose disease incidence (DI) and severity (SS).

Phenotype	Group range description
**Resistant**	= no anthracnose symptoms (0% DI and SS on plant canopy)
**Strongly tolerant**	= DI up to 9.0% or SS less than class 1.5 (from 1 to 2.0 class)
**Tolerant**	= DI more than 9.0% (up to 19%) or SS more than class 2.0 (from 1.5 to 3.0 class)
**Moderately tolerant**	= DI more than 19.0% (up to 40%) or SS more than class 3.0 (from 1.75 to 3.5 class)
**Moderately sensitive**	= DI more than 40% (up to 55%) or SS more than class 3.5 (from 2.75 to 4.25 class)
**Sensitive**	= DI more than 55% (up to 85%) or SS more than class 4.25 (from 3.5 to 5.75 class)
**Highly sensitive**	= DI more than 85% (up to 100%) or SS more than class 5.75 (from 4.0 to 7.0 class)

**Table 2:** Mango cultivars grouped for susceptibility phenotype to anthracnose infections and relative DI and severity (SS).

Phenotype	Cultivar ^a^	DI (%) ^b^	SS (0-to-12 scale) ^b^
**Resistant**	No cultivar	-	-
**Strongly tolerant**	Lita, Natalina, Ourinho, *Mallika,* Nam Dok Mai, Alfa, Heidi, Manilla, Ouroporanga, Winter, Nandoca Arroxeada	5.25 ± 0.69	1.59 ± 0.14
**Tolerant**	Sensação, Coração De Boi Escalope, Carlotinha, Iac 122, *Kent*, Irwin, Juazeiro, Roxa, Espada Valentin, Surpresa, Parvin	12.92 ± 0.84	2.11 ± 0.12
**Moderately tolerant**	Pequi, Itamaracá, Carabao, Umbigo, Imbú, Coração De Boi Barbosa, Espada Ouro, Votupá, Santo Antônio, *Tommy Atkins*, Ômega, Iac 136, Apple, Coquinho, Tinfan, Haden 2h, *Haden,* Ias Haden, Extrema, Neldica, Rosa Vila, Mamão, Fafà	27.93 ± 1.32	2.73 ± 0.10
**Moderately sensitive**	Lira, Espada, Carlota, Batista, Iac 105 Palmeiras, Mastruz, De Cheiro, Ubá Juste, Van Dike, *Kensington Pride,* Mabrooca, Pope, Castro, Sabina Fronteira, Bourbon Do Cláudio, Bourbon, Bourbon Vermelha, Espada Stahl	45.46 ± 1.75	3.54 ± 0.10
**Sensitive**	Dura, Fiapo, Imperial, Governadora, Torrinha, Coração De Boi, Gioana, Rosa Astolfo Dutra, Pingo D’ouro, Vovó, Sabina Léo, Espada Itápolis, *Keitt*	64.81 ± 2.37	4.58 ± 0.16
**Highly sensitive**	*Ubá,* Correio, Aroeira, Rosa, Sapatinho, Amarelinha Da Sementeira, *Palmer,* Quinzenga, Espada Perdões, Roxinha Da Sementeira	96.63 ± 2.08	5.79 ± 0.29

^a^ An amount of 86 mango cultivars was monitored in the Brazilian Mango Germplasm Collection at the Sementeira Farm, Universidade Federal de Viçosa. ^b^ Data are means of disease parameters ± standard error of the mean (SEM) of all mango cultivars included in each phenotype group.

## References

[1] Altendorf S. (2017). Major Tropical Fruits Market Review.

[2] Mitra S.K. (2016). Mango production in the world: Present situation and future prospect. Acta Hortic..

[3] Pott I., Marx M., Neidhart S., Muhlbauer W., Carle R. (2003). Quantitative determination of b-carotene stereoisomers in fresh, dried, and solar dried mangoes (*Mangifera indica* L.). J. Agric. Food Chem..

[4] Nadeem M., Imran M., Khalique A. (2016). Promising features of mango (*Mangifera indica* L.) kernel oil: A review. J. Food Sci. Technol..

[5] Arauz L.F. (2000). Mango anthracnose: Economic impact and current options for integrated management. Plant Dis..

[6] Akem C.N. (2006). Mango anthracnose disease: Present status and future research priorities. Plant Pathol. J..

[7] Sutton B.C. (1980). The Coelomycetes: Fungi Imperfecti with Pycnidia, Acervuli and Stromata.

[8] Sutton B.C., Bailey J.A., Jeger M.J. (1992). The genus *Glomerella* and its anamorph *Colletotrichum*. Colletotrichum: Biology, Pathology and Control.

[9] Cannon P.F., Damm U., Johnston P.R., Weir B.S. (2012). *Colletotrichum*-current status and future directions. Stud. Mycol..

[10] Damm U., Cannon P.F., Woudenberg J.H.C., Crous P.W. (2012). The *Colletotrichum acutatum* species complex. Stud. Mycol..

[11] Damm U., Cannon P.F., Woudenberg J.H.C., Johnston P.R., Weir B.S., Tan Y.P., Shivas R.G., Crous P.W. (2012). The *Colletotrichum boninense* species complex. Stud. Mycol..

[12] Damm U., Cannon P.F., Liu F., Barreto R.W., Guatimosim E., Crous P.W. (2013). The *Colletotrichum orbiculare* species complex: Important pathogens of field crops and weeds. Fungal Divers..

[13] Damm U., O’Connell R.J., Groenewald J.Z., Crous P.W. (2014). The *Colletotrichum destructivum* species complex—Hemibiotrophic pathogens of forage and field crops. Stud. Mycol..

[14] Weir B.S., Johnston P.R., Damm U. (2012). The *Colletotrichum gloeosporioides* species complex. Stud. Mycol..

[15] Cai L., Hyde K.D., Taylor P.W.J., Weir B.S., Waller J.M., Abang M.M., Zhang J.Z., Yang Y.L., Phoulivong S., Liu Z.Y. (2009). A polyphasic approach for studying *Colletotrichum*. Fungal Divers..

[16] Hyde K.D., Cai L., Cannon P.F., Crouch J.A., Crous P.W., Damm U., Goodwin P.H., Chen H., Johnston P.R., Jones E.B.G. (2009). *Colletotrichum*-names in current use. Fungal Divers..

[17] Ismail A.M., Cirvilleri G., Yaseen T., Epifani F., Perrone G., Polizzi G. (2015). Characterisation of *Colletotrichum* species causing anthracnose disease of mango in Italy. J. Plant Pathol..

[18] Jayawardena R.S., Hyde K.D., Damm U., Cai L., Liu M., Li X.H., Zhang W., Zhao W.S., Yan J.Y. (2016). Notes on currently accepted species of *Colletotrichum*. Mycosphere.

[19] Guarnaccia V., Groenewald J.Z., Polizzi G., Crous P.W. (2017). High species diversity in *Colletotrichum* associated with citrus diseases in Europe. Persoonia.

[20] Sharma G., Maymon M., Freeman S. (2017). Epidemiology, pathology and identification of *Colletotrichum* including a novel species associated with avocado (*Persea americana*) anthracnose in Israel. Sci. Rep..

[21] Lima N.B., De A., Batista M.V., De Morais M.A., Barbosa M.A.G., Michereff S.J., Hyde K.D., Câmara M.P.S. (2013). Five *Colletotrichum* species are responsible for mango anthracnose in northeastern Brazil. Fungal Divers..

[22] Sharma G., Kumar N., Weir B.S., Hyde K.D., Shenoy B.D. (2013). The ApMat marker can resolve *Colletotrichum* species: A case study with *Mangifera indica*. Fungal Divers..

[23] Udayanga D., Manamgoda D.S., Liu X., Chukeatirote E., Hyde K.D. (2013). What are the common anthracnose pathogens of tropical fruits?. Fungal Divers..

[24] Vieira W.A.S., Michereff S.J., de Morais M.A., Hyde K.D., Câmara M.P.S. (2014). Endophytic species of *Colletotrichum* associated with mango in northeastern Brazil. Fungal Divers..

[25] Freeman S., Katan T., Shabi E. (1998). Characterization of *Colletotrichum* species responsible for anthracnose diseases of various fruits. Plant Dis..

[26] Ploetz R.C., Prakash O., Litz R.E. (1997). Foliar, floral and soilborne diseases. The Mango: Botany, Production and Uses.

[27] Tarnowski T. (2009). Using Molecular Analysis to Investigate Phylogenetic Relationships in Two Tropical Pathosystems: Witches’ Broom of Cacao, Caused by *Moniliophthora perniciosa*, and Mango Anthracnose, Caused by *Colletotrichum* spp.. Ph.D. Thesis.

[28] Sharma G., Gryzenhout M., Hyde K.D., Pinnaka A.K., Shenoy B.D. (2015). First report of *Colletotrichum asianum* causing mango anthracnose in South Africa. Plant Dis..

[29] Honger J.O., Offei S.K., Oduro K.A., Odamtten G.T., Nyaku S.T. (2014). Identification and species status of the mango biotype of *Colletotrichum gloeosporioides* in Ghana. Eur. J. Plant Pathol..

[30] James R.S., Ray J., Tan Y.P., Shivas R.G. (2014). *Colletotrichum siamense*, *C. theobromicola* and *C. queenslandicum* from several plant species and the identification of *C. asianum* in the Northern Territory, Australia. Australas. Plant Dis. Notes.

[31] Krishnapillai N., Wilson Wijeratnam R.S. (2014). First report of *Colletotrichum asianum* causing anthracnose on Willard mangoes Sri Lanka. New Dis. Rep..

[32] Mo J., Zhao G., Li Q., Solangi G.S., Tang L., Guo T., Huang S., Hsiang T. (2018). Identification and characterization of *Colletotrichum* species associated with mango anthracnose in Guangxi, China. Plant Dis..

[33] Prihastuti H., Cai L., Chen H., McKenzie E.H.C., Hyde K.D. (2009). Characterization of *Colletotrichum* species associated with coffee berries in northern Thailand. Fungal Divers..

[34] Lima N.B., Lima W.G., Tovar-Pedraza J.M., Michereff S.J., Câmara M.P.S. (2015). Comparative epidemiology of *Colletotrichum* species from mango in northeastern Brazil. Eur. J. Plant Pathol..

[35] Shivas R.G., Tan Y.P., Edwards J., Dinh Q., Maxwell A., Andjic V., Liberato J.R., Anderson C., Beasley D.R., Bransgrove K. (2016). *Colletotrichum* species in Australia. Australas. Plant Path..

[36] Tivoli B., Baranger A., Avila C.M., Banniza S., Barbetti M., Chen W., Davidson J., Lindeck K., Kharrat M., Rubiales D. (2006). Screening techniques and sources of resistance to foliar diseases caused by major necrotrophic fungi in grain legumes. Euphytica.

[37] Litz R.E. (2009). The Mango: Botany, Production and Uses.

[38] Delgado P.M.H., Aranguren M., Reig C., Galván D.F., Mesejo C., Fuentes A.M., Saúco V.G., Agustí M. (2011). Phenological growth stages of mango (*Mangifera indica* L.) according to the BBCH scale. Sci. Hortic..

[39] Tu J.C. (1986). A detached leaf technique for screening beans (*Phaseolus vulgaris* L.) in vitro against anthracnose (*Colletotrichum lindemuthianum*). Can. J. Plant Sci..

[40] Iwaro A.D., Sreenivasan T.N., Umaharan P. (1997). Foliar resistance to *Phytophthora palmivora* as an indicator of pod resistance in *Theobroma cacao*. Plant Dis..

[41] Bigirimana J., Höfte M. (2001). Bean anthracnose: Inoculation methods and influence of plant stage on resistance of *Phaseolus vulgaris* cultivars. J. Phytopathol..

[42] Monteon-Ojeda A., Pérez-Rodriguez A., Mora-Aguilera J.A., Sandoval-Islas J.S., De-León-Garciá-De-Alba C., Hernández-Castro E., Vásquez-López A. (2017). Evaluation of tolerance to vegetative anthracnosis of new mango germplasms in Mexico. Trop. Subtrop. Agroec..

[43] Adikshita, Sharma I.M., Sharma M. (2018). Screening of mango germplasm under natural epiphytotic conditions against anthracnose (*Colletotrichum gloeosporioides*). Indian Phytopathol..

[44] Giblin F.R., Tan Y.P., Mitchell R., Coates L.M., Irwin J.A.G., Shivas R.G. (2018). *Colletotrichum* species associated with pre-and post-harvest diseases of avocado and mango in eastern Australia. Australas. Plant Path..

[45] Peterson R.A., Chaplin G.R. (1986). Mango diseases. Proceedings of the CSIRO 1st Australian Mango Research Workshop.

[46] Sharma I.M., Badiyala S.D. (1998). Screening of mango cultivars for susceptibility to *Colletotrichum gloeosporioides* during different seasons. Indian Phytopathol..

[47] Pernezny K., Ploetz R. (2000). Some Common Diseases of Mango in Florida.

[48] Dinh S.Q., Chongwungse J., Pongam P., Sangchote S. (2003). Fruit infection by *Colletotrichum gloeosporioides* and anthracnose resistance of some mango cultivars in Thailand. Australas. Plant Path..

[49] Donald I. (2006). Constitutive Alk(en)ylresorcinols and resistance to postharvest disease in mango (*Mangifera indica* L.). Ph.D. Thesis.

[50] Lei X.T., Zhao Y.L., Yao Q.S., He Y.B., Sun G.M., Ma W.H., Zhan R.L. (2006). Identification and analysis of the resistance of different mango cultivars to anthracnose (*Colletotrichum gloeosporioides*). J. Fruit Sci..

[51] Bhagwat R.G., Mehta B.P., Patil V.A., Sharma H. (2015). Screening of cultivars/varieties against mango anthracnose caused by *Colletotrichum gloeosporioides*. Int. J. Environ. Agric. Res..

[52] O’Donnell K., Cigelnik E. (1997). Two divergent intragenomic rDNA ITS2 types within a monophyletic lineage of the fungus *Fusarium* are non orthologous. Mol. Phylogenet. Evol..

[53] Glass N.L., Donaldson G.C. (1995). Development of primer sets designed for use with the PCR to amplify conserved genes from filamentous ascomycetes. Appl. Environ. Microbiol..

[54] White T.J., Bruns T., Lee S., Taylor J.W., Innis M.A., Gelfand D.H., Sninsky J.J., White T.J. (1990). Amplification and direct sequencing of fungal ribosomal RNA genes for phylogenetics. PCR Protocols: A Guide to Methods and Applications.

[55] Crous P.W., Groenewald J.Z., Risede J.M., Hywel-Jones N.L. (2014). *Calonectria* species and their *Cylindrocladium* anamorphs: Species with sphaeropedunculate vesicles. Stud. Mycol..

[56] Kumar S., Stecher G., Li M., Knyaz C., Tamura K. (2018). MEGA X: Molecular evolutionary genetics analysis across computing platforms. Mol. Biol. Evol..

[57] Tamura K. (1992). Estimation of the number of nucleotide substitutions when there are strong transition-transversion and G+C-content biases. Mol. Biol. Evol..

[58] Tamura K., Nei M. (1993). Estimation of the number of nucleotide substitutions in the control region of mitochondrial DNA in humans and chimpanzees. Mol. Biol. Evol..

[59] Schneider C.A., Rasband W.S., Eliceiri K.W. (2012). NIH Image to ImageJ: 25 years of image analysis. Nat. Methods.

[60] Piccirillo G., Carrieri R., Polizzi G., Azzaro A., Lahoz E., Fernández-Ortuño D., Vitale A. (2018). In vitro and in vivo activity of QoI fungicides against *Colletotrichum gloeosporioides* causing fruit anthracnose in *Citrus sinensis*. Sci. Hortic..

[61] Shah D.A., Madden L.V. (2004). Nonparametric analysis of ordinal data in designed factorial experiments. Phytopathology.

